# Researching social innovation: is the tail wagging the dog?

**DOI:** 10.1186/s40249-019-0616-7

**Published:** 2020-01-13

**Authors:** Emma L. M. Rhule, Pascale A. Allotey

**Affiliations:** United Nations University – International Institute for Global Health, Kuala Lumpur, Malaysia

**Keywords:** Social innovation, Health systems, Mixed methods, Participation, Community engagement, Policymaker, Implementation research

## Abstract

**Background:**

Social Innovation in health initiatives have the potential to address unmet community health needs. For sustainable change to occur, we need to understand how and why a given intervention is effective. Bringing together communities, innovators, researchers, and policy makers is a powerful way to address this knowledge gap but differing priorities and epistemological backgrounds can make collaboration challenging.

**Main text:**

To overcome these barriers, stakeholders will need to design policies and work in ways that provide an enabling environment for innovative products and services. Inherently about people, the incorporation of community engagement approaches is necessary for both the development of social innovations and accompanying research methodologies. Whilst the 'appropriate' level of participation is linked to intended outcomes, researchers have a role to play in better understanding how to harness the power of community engagement and to ensure that community perspectives form part of the evidence base that informs policy and practice.

**Conclusions:**

To effectively operate at the intersection between policy, social innovation, and research, all collaborators need to enter the process with the mindset of learners, rather than experts. Methods – quantitative and qualitative – must be selected according to research questions. The fields of implementation research, community-based participatory research, and realist research, amongst others, have much to offer. So do other sectors, notably education and business. In all this, researchers must assume the mantel of responsibility for research and not transfer the onus to communities under the guise of participation. By leveraging the expertise and knowledge of different ecosystem actors, we can design responsive health systems that integrate innovative approaches in ways that are greater than the sum of their parts.

## Background

Communities and social innovators develop and drive solutions to challenges, empowered by the desire for change. In health, as in many other sectors, innovations have been spurred in response to problems which have been ignored or inadequately addressed by formal systems. Social innovations in health have been further promoted through financial incentives offered through initiatives such as the Grand Challenges [1] or by those offered through global health research funding bodies. However, a limited understanding of the components of an innovation that underpin its success (or failure) can limit the ability to learn from its implementation, or to replicate or scale it up to other communities and populations. This presents a missed opportunity for health systems that could draw lessons for active engagement with communities in designing and implementing health services and interventions that are sustained in communities. Collaborations between health researchers and policy makers, social innovators, and communities have the potential to address this knowledge deficit.

Along these lines, a recent forum brought together social innovators, researchers, and other key stakeholders to nurture collaboration and establish a research agenda. The forum provided the opportunity for rich, in-depth discussion and engagement and largely, achieved the planned objectives [2]. However, a number of fault lines were also revealed. These primarily stemmed from the differing priorities and perceived notions of success of the range of stakeholders. Social innovators and communities were engrossed in the day-to-day activities required to meet health needs, implement programmes, and create the change they want to see and live. For researchers, adherence to methodological rigour was paramount to ensure that any evidence generated could fit into scientific paradigms for reproducibility. From the perspective of health policy makers, social innovations provided an opportunity to devolve responsibility to service providers that were more acceptable to the community, but also raised the challenge of sustainable financing. Attempts to instrumentalise the utility of social innovation for the various stakeholders risked losing the very values that underpinned and motivated social innovation.

In this paper, we explore the features of social innovation that contribute to its success and unpack the challenges of undertaking research within the context of community-driven social innovation. We posit the notion that research needs to be in service to the community and to social innovation, and therefore requires innovation in approaches and design in order to balance rigour against the realities of working with, and responding to, community-driven demand. We also present suggestions for practices that researchers and social innovators could adopt to build and maintain mutually beneficially collaborations.

## Conceptualising social innovation in health

Phills et al. [3] describe social innovation as “a novel solution to a social problem that is more effective, efficient, sustainable, or just than existing solutions and for which the value created accrues primarily to society as a whole rather than private individuals”. The arenas of healthcare, education, and environmental sustainability have been ripe areas for innovation [4]. Whilst the creation of locally designed solutions is not a new phenomenon, technological advancements and increasing globalisation have facilitated major leaps in the scale and impact of solutions.

Social innovations are wide ranging, encompassing products, services, behavioural practices, and models or policies. Many are a combination of these. Innovations do not have to be new inventions, or new to the world, but their deployment should be novel either to the beneficiary group or in the way in which they are applied. For example, Riders for Health [5], uses a very familiar product — a motorcycle — to address major challenges in last-mile health delivery, particularly for rural communities. Working with ministries of health, Riders for Health teaches community health workers how to ride motorbikes, as well as basic maintenance and repair skills. Riders also provide transportation services for both medical necessities and people. Now operational in eight countries, each country team operates as an independent organisation with the flexibility to adapt to local contexts and needs. ICHAPP, a Brazilian Indigenous Community Health Agent Professionalisation Programme [5], aims to improve health care services in remote indigenous communities by blending traditional practices and cultural beliefs with biomedical approaches. As well as recognising communities’ contextual realities, the accompanying training and certification programme for indigenous community health agents has enabled them to receive a salary for their work. In both these cases, innovations were developed with and adapted to the needs of beneficiary communities, illustrating the social orientation of social innovation in ‘both ends and means’ [6].

## Community engagement and social innovation

Community engagement has been conceptualised in a number of different ways from Arnstein’s seminal Ladder of Participation [7] to Reed’s more recent Wheel of Participation [8]. They all seek to address three common features of engagement: 1) **Why?** What are the motivations for engagement, be they pragmatic (better outcomes), normative (an expectation that stakeholders/publics should participate in major decisions that affect them), or to enhance trust in decision-making processes?; 2) **How** is the engagement being carried out? Methods are often presented along a continuum of engagement from communicating information, consulting for feedback, to collaboration and co-production; and, 3) **Who** is the initiating party? Is the engagement being driven from the bottom-up, or is top-down?

Historically, the underlying rationale for adopting community engagement approaches has been inherently value-laden. Arnstein’s ‘ladder of citizen participation’ [7] presents the degree of co-operation and participation between different actors with two rungs of non-participation at the base, followed by three rungs of tokenism, and finally three levels of citizen power. The visual of a ladder combined with Arnstein’s focus on the potential power (im)balances between different stakeholders implies a scale of ‘bad’ to ‘good’ engagement. This perspective is rooted in the deployment of community engagement in situations where power dynamics are unequal and community mobilisation serves as a form of activism. Social innovation, by its very nature, is accommodating of bottom-up endeavours. However, it would be naïve to imply that power imbalances do not occur. They may be driven by factors including unequal availability of resources, access, and expertise. When the type or number of stakeholders engaged in social innovation expands, especially with the introduction of experts, e.g. researchers, or influential stakeholders, power dynamics are likely to come into play. However, there are many ways in which communities and other stakeholders can engage, and methods to deploy, that aim to flatten power hierarchies. These range from individual reflexive practices in which participants consider their own biases and privileges, to facilitation techniques that provide space for debate and accommodate different modes of participation. Depending on the nature of the project and the needs, wants, and other commitments of communities, ‘shallower’ levels of engagement, such as consultation’ may be appropriate or even desired.

In response to a values-based approach, and in an attempt to mainstream community engagement, practitioners have increasingly presented utilitarian arguments for the benefits of engagement, including increased initiative effectiveness and sustainability, and economic benefits [9]. This tension continues to be visible in the interactions between social innovators and researchers. An argument grounded in rights or values is sufficient for social innovation because ultimately, it’s about people. The challenge for health researchers is that these aren’t things that are typically measured. Thus, there is a need to balance the intrinsic value and instrumental benefits of community engagement approaches to produce positive health outcomes while managing the risk of engagement becoming just a tick-box exercise.

## Research challenges at the intersection of social innovation and health

Research in the context of social innovation in health is trying to find solutions and approaches that meet community needs *and* make a unique contribution to the development of resilient health systems. However, what ‘success’ looks like to the different stakeholders involved can vary. Operationally relevant results, i.e. data that guide intervention iteration, increase efficiencies, and maximise impact, are useful for innovation implementers. For researchers, measures of health outcomes are key while for policy makers, such research is most useful when it addresses not only how and why an intervention works, but how to implement it sustainably. This can present challenges for researchers used to methods that rely on an intervention having fixed and/or controlled variables, such as the ‘gold standard’ of medical research, randomised controlled trials. The growth of the field of implementation research (IR) has started to address these gaps. Implementation research seeks to understand what, why, and how interventions work in a given context. With its origins in several different research fields, IR has an inherently mixed methods approach to study design as well as being sufficiently flexible to account for and adapt to changes in the intervention being implemented [10].

The focus on users, whether communities, policymakers etc., is another strength of IR, moving research beyond a focus on the production of knowledge. It means that a collaborative approach to research design is integral to any project and requires that community and broader stakeholder engagement approaches are treated as being on a par with research methodologies in terms of importance. This can present challenges, from a lack of knowledge — the approaches are not a standard part of the curriculum for health researchers — to a lack of methodological trust [11]. Fields of research practice that are grounded in community and participatory approaches can provide insights into ways of working. For example, realist research approaches [12] seek to understand how the outcomes of complex interventions and programmes are impacted by context. This requires a greater engagement by researchers in understanding the issues from a grassroots perspective. Similarly, methods from participatory action research [13] and community-based participatory research are a valuable additions to a researcher’s toolkit [14]. In each of these approaches, co-design is essential. Beyond the sciences, a lot can be learnt from other sectors including design research, business, and education. There is much to gain by venturing beyond the boundaries of individual epistemological backgrounds.

In many cases, “we have learned to create the small exceptions that can change the lives of hundreds. But we have not learned how to make the exceptions to the rule to change the lives of millions” [15]. Adoption of innovations by ministries of health and incorporation into health systems is a powerful approach for providing long term sustainability, and potential to scale, for innovations that improve lives. For implementation uptake to be successful an adequate understanding of what’s working is needed. This includes identifying the critical components, the human and financial resources required, and economic costings that provide information on return on investment (ROI) or service delivery savings [16]. This data is most useful when it also addresses how a given programme fits into a broader policy portfolio or aligns to political priorities. Lacking this suite of information hinders uptake and delays or denies communities access to effective services.

## Bringing it all together

Chipatala Cha Pa Foni (CCPF): Health Centre by Phone is a mHealth programme in Malawi that provides health advice over the phone. The initial idea was the combination of two winning submissions to an innovation competition funded by the Bill and Melinda Gates Foundation. Launched in 2011 by Non-Governmental Organisation (NGO) VillageReach, the concept was further developed in collaboration with traditional leaders, community health workers, and district health staff from the Malawian Ministry of Health. A data-based approach was embedded from the outset, with the implementing organisation working in partnership with a research organisation, Invest in Knowledge, who conducted the project evaluation [17]. This combination of innovators, target communities, policymakers, and researchers has resulted in the refinement of the idea, an expanded focus from maternal and child health to general health advice, service increase from one to 28 districts, and transition of the service from the NGO to the national government [5].

A collaboration with health researchers from the outset of an innovation is the exception rather than the rule. That doesn’t mean that a research component cannot subsequently be integrated, however in doing so, researchers must retain the mantel of responsibility for research and not transfer the onus to communities and innovators under the guise of participation. When exploring perceived barriers to collaborating with researchers, social innovators mentioned that many researchers expected them to take on the data collection elements of research which they lacked the capacity (time, resource, and skills) for. Whilst collaborations can present good opportunities for skills transfer and capacity development within communities, they also provide an opportunity for Master’s and PhD students to collect data while gaining experience of health innovation in communities. This could be a way to balance the tension between adhering to ‘standards of evidence’ and not extinguishing innovation or the enthusiasm of community-generated projects.

## Conclusions

Partnerships between social innovators, communities, policy makers and researchers can leverage the experience and expertise of each to gain and advance vital knowledge. To be effective brokers at this intersection, researchers need to be willing to enter the process as learners, not just experts. Too often we think about participatory models of engagements from a single perspective: the expert researcher coming in and engaging communities with their work. By adopting a more holistic approach, valuing each stakeholder as a holder of expertise as well as a recipient of new information, and emphasising the co-creation of knowledge and convergence of goals, we improve the chances of long-term behaviour change.

The ultimate goal of achieving good health and wellbeing [18] unifies communities, innovators, researchers and policymakers. Combined with a global push towards ‘people-centred healthcare’ [19], the flourishing number of social innovations in the delivery of health services driven directly by communities, grassroots organisations, and social innovators should not be surprising. To achieve meaningful progress, all stakeholders are going to have to come together with no one element dominating. By leveraging the expertise and knowledge of different ecosystem actors, we can design responsive health systems that integrate innovative approaches and take us closer to achieving ‘health for all’.

## Data Availability

Not applicable.

## Abbreviations

CPPF: Chipatala Cha Pa Foni
ICHAPP: Indigenous Community Health Agent Professionalisation Programme
IR: Implementation
NGO: Non-Governmental Organisation
ROI: Return on Investment
SIHI: Social Innovation in Health Initiative
